# Medical Expenditure Differences Between Income Levels Among US Adults With Diabetes

**DOI:** 10.5888/pcd22.250153

**Published:** 2025-08-07

**Authors:** Yu Wang, Hui Shao, Elizabeth Bigman, Christopher Holliday, Ping Zhang

**Affiliations:** 1 Division of Diabetes Translation, Centers for Disease Control and Prevention, Atlanta, Georgia; 2 Hubert Department of Global Health, Rollins School of Public Health, Emory University, Atlanta, Georgia

## Abstract

**Introduction:**

Significant differences exist in the risk of diabetes and diabetes-related complications by income level in the United States. We assessed 1) to what extent medical expenditures in total and by health service type differ by income levels, and 2) how demographic and socioeconomic factors and health status are associated with these differences.

**Methods:**

Data from the 2017 through 2021 Medical Expenditure Panel Survey were analyzed to estimate annual per-person medical expenditures for adults with diabetes. These expenditures were categorized by service type (inpatient, outpatient, prescription, home health care services, emergency department, or other) and compared across income groups based on the federal poverty level (FPL): poor (<125% FPL), low (125% to <200% FPL), middle (200% to <400% FPL), and high (≥400% FPL). One-way analysis of variance was used to test group differences, and a regression-based decomposition identified factors driving expenditure disparities. All expenditures were adjusted to 2021 US dollars.

**Results:**

Mean total medical expenditures were significantly higher for the poor-income group compared with the low-income, middle-income, and high-income groups, though no significant differences were observed among the latter 3 groups. Prescription drugs and home health care services in the poor-income group accounted for most of this difference. Key factors associated with the higher expenditures in this group included elevated disability rates, poorer physical health status, and dual Medicaid–Medicare coverage.

**Conclusion:**

Adults with diabetes from the poorest households incurred the highest medical expenditures, largely driven by poor physical health and higher rates of disability. Reducing disability and improving health outcomes for this group may help lower their medical expenses.

**Summary**

**What is already known on this topic?**

People from low-income families are disproportionately affected by diabetes and have higher medical expenditures due to greater health care needs. They also face significant barriers to accessing quality care.

**What is added by this report?**

We examined the relationship between income and medical spending. Adults from households with incomes below 125% of the federal poverty level had significantly higher medical expenditures than those from higher income households. This difference was largely driven by increased spending on prescriptions and home health care services. High rates of disability and poor physical health among the low-income group contributed to these elevated costs.

**What are the implications of our findings?**

Efforts to improve the health of adults with diabetes from low-income households may help lower overall health care expenditures.

## Introduction

Significant differences exist in the risk of diabetes and diabetes-related complications by income level in the US. People with lower incomes are disproportionately affected by diabetes (1); adults with a family income below the federal poverty level (FPL) have the highest prevalence of diabetes. In 2019 through 2021, for example, 13.1% of people having a family income below the FPL had diabetes compared with 5.1% of people with a family income of 500% or greater of the FPL (2). Low-income people with diabetes also face more challenges with diabetes management, with a higher rate of uncontrolled hemoglobin A_1c_, high blood pressure, and high lipid levels (3–5), as well as higher rates of diabetes-related complications and premature death (6,7) and barriers to quality care (8,9).

For people with diabetes, the relationship between income and medical spending is complex. Low-income people with diabetes may have poorer health status (10), requiring more health services leading to higher expenditures. Yet their barriers to accessing quality care or new treatments could result in underuse of services and lower expenditures (8). Benefit coverage of insurance and social programs for poor people could also affect their spending. In some countries, people with high incomes have high medical expenditures (11), while in other countries people with the lowest income have the highest medical expenditures (12,13). The pattern, magnitude, and factors associated with income-related differences in medical expenditures in people with diabetes have not been assessed in the US.

In this study, we examined differences in medical expenditures by income level among US adults with diabetes. We hypothesized that US adults with diabetes from low-income households may incur higher annual per-person medical expenditures than those in higher-income households, potentially due to poorer physical health status, but that the composition of their spending will differ. Specifically, we assessed 1) to what extent medical expenditures in total and by health service type differ by income levels and 2) how demographic and socioeconomic factors and health status are associated with these differences.

## Methods

### Data source and study population

We used data from the 2017 through 2021 Medical Expenditure Panel Survey (MEPS), Household Component. MEPS is a nationally representative household survey of the US civilian noninstitutionalized population that contains information about health conditions, health care service usage, and expenditures (14). The study population included adults aged 18 years or older with self-reported diabetes. We identified people with diabetes by the question, “Have you ever been told by a doctor or other health professional that you had diabetes?” Adults who answered yes to this question were included in the analysis. We pooled 5 years of data to achieve a sufficient sample size for the study.

### Measures

#### Income groups

We defined 4 income categories based on FPL. FPL is a measurement that describes the minimum income a person or a family needs to pay for bare living essentials specific to family size and is updated by the US government yearly (15). Percentage of FPL was calculated by dividing the total family income with the official FPL for that family size; this calculation is often used to decide whether the income level of the person or family qualifies for certain federal benefits and programs (15). Four household income levels were defined: poor income (<125% FPL), low income (125% to <200% FPL), middle income (200% to <400% FPL), and high income (≥400% FPL). We used 125% of the FPL as the threshold for poor income because it is an income that would qualify a person or a family for many federal support programs, including Medicaid in most states, Supplemental Nutrition Assistance Program, and legal aid. Low income was defined as 125% to less than 200% of the FPL to capture households that are still economically vulnerable and qualify for some federal support programs, including childcare subsidies and the Children’s Health Insurance Program. At 400% of the FPL, income is where people would not receive federal support on health insurance premiums under the Affordable Care Act and, thus, is defined as the starting point of high income.

#### Variables

The primary outcome was total annual medical expenditures per person, which is the sum of the direct payments for care provided during the year, including out-of-pocket payments and payments by private insurance, Medicaid, Medicare, or other sources. We also examined annual per-person expenditure components by type of health service: inpatient, outpatient, prescription, home health care services, emergency department, or others (eg, glasses or contact lenses, ambulance, disposable supplies, long-term use of equipment). Differences were defined as the difference in average annual per-person expenditures between each pair of income categories by subtracting the average expenditure for adults from the higher income group from the average expenditure for adults from the lower income group. All expenditures were adjusted to 2021 US dollars by using the Personal Consumption Expenditures index (16).

We used a regression-based decomposition method to examine the association of differences in each pair of income categories where their difference in medical expenditures was significant with demographic and socioeconomic factors and health status (17). The factors included in the regression models were age group (18–44 y, 45–54 y, 55–64 y, ≥65 y), sex (female, male), race and ethnicity (non-Hispanic White, non-Hispanic Black, Hispanic, other [including American Indian or Alaska Native, Asian, Native Hawaiian or Pacific Islander, or multiple races]), insurance (Medicaid–Medicare dual coverage, Medicare only or with private, Medicaid only, private insurance only, uninsured, other), education (less than a college degree, college degree or higher), disability status (yes, no), perceived physical health (good, poor), and perceived mental health (good, poor). Disability was defined by any self-reported daily living, functional, or activity limitations.

### Statistical analyses

Characteristics of people in the 4 income groups were summarized as number and percentage with standard error (SE) for categorical variables and mean with SE for continuous variables. Differences in demographic and socioeconomic factors and health status between income groups were compared using χ^2^ tests for categorical variables and *t* tests for continuous variables. We used the 1-way analysis of variance (ANOVA) method to test if expenditure differences between adults from poor versus low, poor versus middle, poor versus high, low versus middle, low versus high, and middle versus high household income groups were statistically different. A *P* value of .05 or less was used to define significant differences. All variables were weighted according to the MEPS guideline.

For income groups with significant expenditure differences, we used the Blinder−Oaxaca decomposition method to examine how each demographic and socioeconomic factor and health status variable was associated with the difference (17). Blinder–Oaxaca decomposition is a regression-based method that predicts the mean expenditure of a group by using the mean value of each included factor and its estimated regression coefficient. This method specifies how much of the difference in the mean expenditures between 2 groups is explained by the difference in the mean values of included factors. The remaining discrepancy cannot be explained by factors in the model (18). A factor can contribute positively or negatively to the difference in the mean value of the expenditure between 2 income groups. Elevating factors are defined as factors associated with higher difference; offsetting factors are associated with lower difference. It is possible that the explained difference could be larger than the actual difference, which indicates that there should be an even bigger difference between the groups based on the factors included in the model alone. This discrepancy could be due to limitations in the model or unobserved factors that neutralize the expected effect.

### Sensitivity analysis

We conducted 2 additional analyses. First, we applied the same analytical approach to people without diabetes and compared these results to the corresponding results for people with diabetes to explore the effect of diabetes on expenditure difference. Second, we conducted an analysis of utilization and per-unit expenditure for medical services where the difference in expenditure of this service divided by the difference of the total medical expenditure between income groups was greater than 20%. This analysis aimed to better describe how utilization, either individually or in combination, explained the observed difference.

## Results

The study included 14,227 adults with self-reported diabetes, ranging from 2,741 in 2017 to 3,125 in 2021. Demographic and socioeconomic characteristics and health status differed significantly across the 4 income groups. The adults from poor-income households, compared with those in other income groups, had the highest proportion of young people (aged 18–44 y); women; Black and Hispanic people; people with dual Medicaid–Medicare coverage, Medicare only, or uninsured; people without a college degree; people reporting poor physical or mental health status; and people with a disability (Table 1).

**Table 1:** Characteristics of US Adult Population With Diabetes, by Income Level, 2017–2021 Medical Expenditure Panel Survey^a^

Characteristic	Poor income (<125% FPL)	Low income (125% to <200% FPL)	Middle income (200% to <400% FPL)	High income (≥400% FPL)	*P* value
**Sample size**	4,009	2,386	3,913	3,919	
**Weighted sample**	28,508,850	21,108,144	39,644,109	48,866,689	
**Mean age, y**	61.2 (0.4)	63.4 (0.5)	61.7 (0.4)	61.9 (0.4)	<.001
**Age, %**					
18–44	15.0 (1.0)	14.3 (1.3)	13.7 (1.1)	8.4 (0.8)	<.001
45–54	12.8 (1.0)	10.3 (1.1)	15.5 (1.1)	15.5 (1.3)	
55–64	27.6 (1.2)	20.3 (1.5)	22.3 (1.2)	28.8 (1.4)	
≥65	44.6 (1.5)	55.1 (2.2)	48.4 (1.6)	47.3 (1.7)	
**Sex, %**					
Female	59.4 (1.4)	53.1 (1.8)	47.6 (1.2)	40.9 (1.4)	<.001
Male	40.5 (1.4)	46.9 (1.8)	52.4 (1.2)	59.1 (1.4)	
**Race and ethnicity, %**					
Non-Hispanic White	45.6 (2.1)	53.8 (2.0)	60.2 (1.6)	68.9 (1.5)	<.001
Non-Hispanic Black	22.0 (1.6)	17.3 (1.6)	13.8 (1.0)	11.0 (1.0)	
Hispanic	23.0 (1.9)	20.7 (1.7)	17.5 (1.3)	10.2 (0.9)	
Other^b^	9.4 (1.0)	8.2 (0.8)	8.5 (0.8)	9.9 (0.9)	
**Insurance, %**					
Medicaid–Medicare dual coverage	30.6 (1.3)	17.0 (1.2)	6.7 (0.6)	3.1 (0.6)	<.001
Medicare only or with private	28.7 (1.1)	46.3 (1.7)	45.2 (1.3)	43.8 (1.4)	
Medicaid only	27.4 (1.4)	14.5 (1.2)	7.8 (0.6)	2.4 (0.3)	
Private only	6.3 (0.7)	15.2 (1.1)	35.7 (1.3)	48.0 (1.4)	
Uninsured	5.8 (0.6)	5.6 (0.7)	3.4 (0.4)	1.3 (0.3)	
Other insurance^c^	1.2 (0.3)	1.4 (0.4)	1.2 (0.2)	1.4 (0.3)	
**Education, %**					
College degree or higher	8.7 (0.8)	12.4 (1.3)	19.6 (1.1)	38.7 (1.4)	<.001
**Self-reported health status, %**					
Poor physical health	49.6 (1.1)	37.2 (1.4)	30.2 (1.1)	23.2 (0.9)	<.001
Poor mental health	26.8 (1.0)	17.8 (1.1)	12.1 (0.7)	8.1 (0.6)	<.001
Disability	64.6 (1.4)	54.4 (1.4)	42.3 (0.4)	35.8 (1.2)	<.001

Abbreviation: FPL, federal poverty level.

a Unless otherwise noted, the data are weighted means (standard errors).

b American Indian or Alaska Native, Asian, Native Hawaiian or Pacific Islander, or multiple races.

c Other public insurance and other hospital or physician coverage.

### Differences in medical expenditures

**Figure 1:**
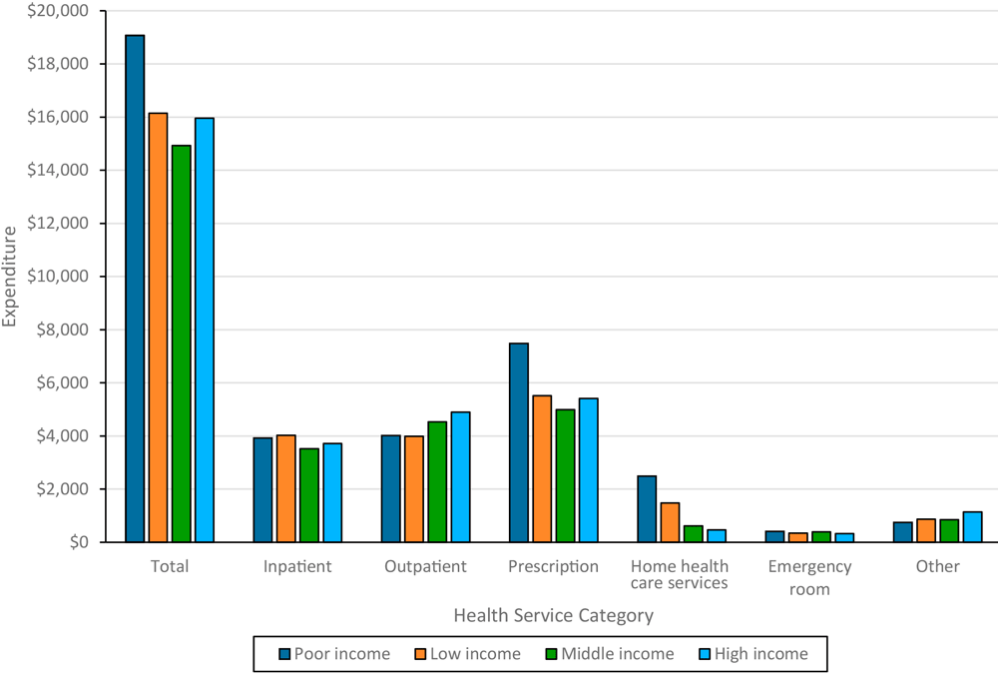
Mean per person per year medical expenditures in US dollars, by income level among people with diabetes, Medical Expenditure Panel Survey, 2017–2021. Income categories were defined based on federal poverty level (FPL), which incorporates both household income and size: poor income (<125% FPL), low income (125% to <200% FPL), middle income (200% to <400% FPL), and high income (≥400% FPL).

Mean total annual medical per-person expenditures were highest in adults from the poor-income households ($19,071), followed by the low-income ($16,143), high-income ($15,961), and middle-income ($14,930) households (Figure 1). The largest annual per-person expenditure difference, which was between adults from poor-income households and those from the middle-income households, was $4,141 (Table 2). ANOVA showed the expenditures of adults from poor-income households significantly exceeded expenditures in the other 3 groups; other group differences were not significant.

**Table:** 

Income level	Total	Inpatient	Outpatient	Prescription	Home health care services	Emergency room	Other
Poor income	19,071	3,921	4,017	7,481	2,494	409	748
Low income	16,143	4,022	3,991	5,517	1,475	348	866
Middle income	14,930	3,522	4,533	4,989	616	391	850
High income	15,961	3,718	4,897	5,412	471	328	1,141

**Table 2:** Total Mean Per Person Per Year Medical Expenditure (US Dollars) and Comparisons by Health Service Type and Income Level Among the US Adult Population With Diabetes, 2017–2021 Medical Expenditure Panel Survey^a^

Difference	Total	Inpatient	Outpatient	Prescription	Home health care services	Emergency department	Other
**Poor income**	19,071 (767)	3,921 (343)	4,017 (264)	7,481 (379)	2,494 (298)	409 (24)	748 (44)
Poor vs Low	2,928^b^ (926)	−100 (544)	102 (362)	1,964^b^ (383)	1,019^b^ (363)	62 (48)	−118 (83)
Poor vs Middle	4,141^b^ (872)	400 (395)	−546 (382)	2,492^b^ (375)	1,878^b^ (299)	19 (47)	−101 (63)
Poor vs High	3,110^b^ (909)	204 (482)	−875^b^ (339)	2,069^b^ (412)	2,022^b^ (301)	82^b^ (39)	−393^b^ (66)
**Low income**	16,143 (743)	4,022 (438)	3,991 (78)	5,517 (276)	1,475 (216)	348 (42)	866 (77)
Low vs Middle	1,213 (848)	500 (502)	−648 (343)	528 (293)	860^b^ (212)	−43 (56)	16 (88)
Low vs High	181 (994)	304 (535)	−977^b^ (340)	105 (320)	1,004^b^ (234)	20 (48)	−275^b^ (94)
**Middle income**	14,930 (543)	3,522 (239)	4,533 (267)	4,989 (177)	616 (68)	391 (38)	850 (45)
Middle vs High	−1,031 (693)	−196 (382)	−328 (314)	−423 (238)	144 (234)	63 (46)	−291^b^ (58)
**High income**	15,961 (515)	3,718 (316)	4,897 (216)	5,412 (193)	471 (100)	328 (29)	1,141 (45)

a The presented numbers are mean (standard error).

b Significantly different at *P* < .05.

Annual inpatient service expenditures per person ranged from $3,522 (middle-income) to $4,022 (low-income), with no significant differences. Outpatient expenditures ranged from $3,991 (low-income) to $4,897 (high-income), with expenditures for adults from poor-income and low-income households significantly lower than for high-income households. Prescription drug expenditures ranged from $4,989 (middle-income) to $7,481 (poor-income), with expenditures for adults from poor-income households significantly higher than for the others; other differences were not significant. Expenditures for home health care services ranged from $471 (high-income) to $2,494 (poor-income), decreasing with decreasing income, with significant differences between all pairs except middle-income and high-income. Emergency department expenditures ranged from $328 (high-income) to $409 (poor-income), significant only between these 2. Other medical expenditures ranged from $748 (poor-income) to $1,141 (high-income), with adults from high-income households significantly higher than the others (Figure 1; Table 2).

### Decomposition Analysis

#### Total medical expenditures

**Figure 2:**
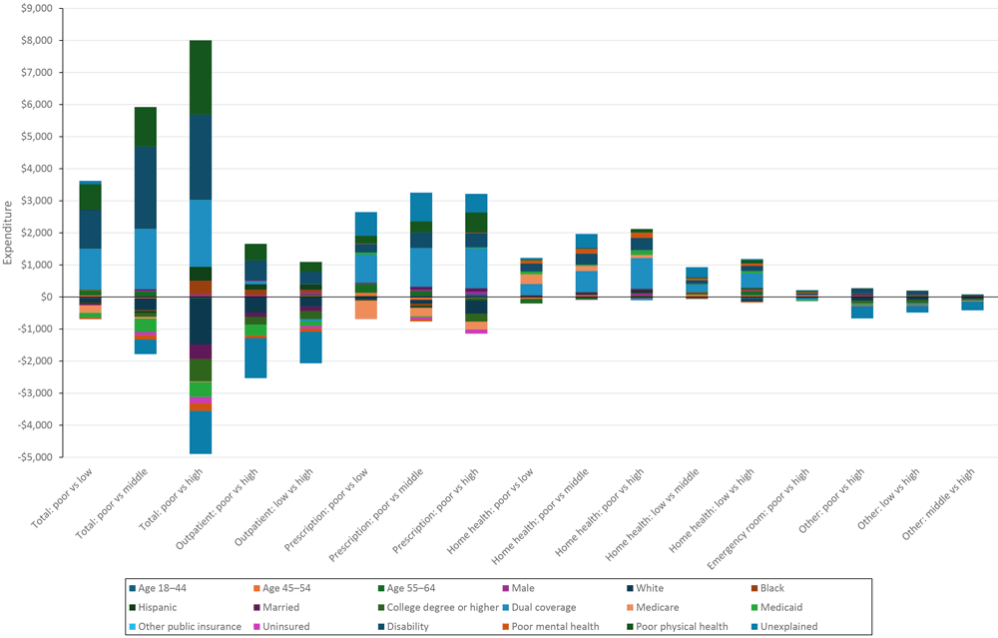
Decomposition results between income groups in total and by health service types, in US dollars, among adults with diabetes, Medical Expenditure Panel Survey, 2017–2021. Each bar represents the total difference in expenditures between the respective income groups, with the label underneath indicating the specific comparison (eg, Outpatient: poor vs high). A positive bar segment means that the factor is associated with increase in the difference in medical expenditures between the income groups. Conversely, a negative bar segment indicates that the factor is associated with decrease in the difference in expenditures. The total bar height (or depth) represents the net difference in medical costs, accounting for both positive and negative contributions. The unexplained portion may include factors not accounted for by the selected variables, representing residual disparities beyond what the model can explain. Income categories were defined based on federal poverty level (FPL), which incorporates both household income and size: poor income (<125% FPL), low income (125% to <200% FPL), middle income (200% to <400% FPL), and high income (≥400% FPL). Abbreviation: Dual coverage, coverage with both Medicaid and Medicare.

For total annual per-person medical expenditures, decomposition analyses were performed on the 3 comparison groups whose differences were significant in the ANOVA models (adults from poor-income households vs adults from each of the other income level households) (Figure 2). Among adults from the poor-income households relative to the low-income households, 99.7% of the difference of the higher expenditure could be explained by the factors included in the model. Significant elevating factors were a higher percentage of Medicaid–Medicare dual coverage (43%), a higher disability rate (41%), and a higher proportion reporting poor physical health (27%). For the poor-income versus middle-income and high-income comparisons, the model predicted an 11% higher difference than was observed, indicating the existence of offsetting factors that were not included our model. Dual coverage, disability, and poor physical health were again the significant elevating factors. For the poor income versus high income comparison, the model predicted a 43% higher difference than was observed, indicating the effect of offsetting factors beyond our model; a lower proportion of the White population, a higher proportion of Black population, and a lower college education rate were significant factors, in addition to Medicaid–Medicare dual coverage, disability, and poor physical health.

**Table:** 

Income group comparison	Total: poor vs low	Total: poor vs middle	Total: poor vs high	Outpatient: poor vs high	Outpatient: low vs high	Prescription: poor vs low	Prescription: poor vs middle	Prescription: poor vs high	Home health: poor vs low	Home health: poor vs middle	Home health: poor vs high	Home health: low vs middle	Home health: low vs high	Emergency room: poor vs high	Other: poor vs high	Other: low vs high	Other: middle vs high
Age 18–44 y	(1)	25	19	16	13	34	35	82	(17)	(14)	(46)	(10)	(77)	0	(13)	(16)	(11)
Age 45–54 y	51	(41)	(7)	(4)	17	98	(88)	(28)	(45)	40	16	79	46	0	2	9(7)	(3)
Age 55–64 y	143	150	(30)	0	41	276	154	(64)	(140)	(54)	29	44	133	1	4	31	8
Male	(23)	29	67	45	74	9	41	86	5	20	72	(5)	27	(2)	27	22	8
White	(129)	(368)	(1,446)	(481)	(293)	(77)	(121)	(422)	5	(13)	6	(30)	(41)	(21)	(101)	(67)	(37)
Black	21	(27)	424	167	84	(15)	(66)	(8)	13	12	1	25	36	10	11	5	(1)
Hispanic	(32)	(58)	431	170	166	(18)	(53)	2	6	12	78	2	41	(4)	17	17	8
Married	(71)	40	(454)	(127)	(146)	21	92	103	22	56	53	(1)	(21)	15	38	11	14
College degree or higher	15	(129)	(695)	(249)	(247)	10	(10)	(246)	2	14	1	5	-13	(6)	(84)	(106)	(55)
Dual coverage	1,263	1,879	2,080	83	(73)	874	1,200	1,244	352	657	962	234	454	(60)	(9)	(29)	(8)
Medicare	(239)	(52)	(17)	0	3	(574)	(270)	(248)	302	162	97	(23)	(28)	30	6	(3)	0
Medicaid	(144)	(417)	(470)	(338)	(143)	66	(31)	22	88	32	145	16	71	(36)	(45)	(28)	(7)
Other public insurance	8	5	6	0	0	0	1	6	1	0	0	(1)	0	0	1	0	0
Uninsured	(4)	(98)	(190)	21	(86)	(3)	(62)	(129)	0	(9)	(19)	0	1	(4)	(16)	(18)	(7)
Disability	1,212	2,568	2,663	645	387	265	492	449	242	349	383	121	158	61	154	99	37
Poor mental health	(49)	(136)	(246)	(84)	(95)	13	(63)	16	102	149	164	56	73	31	(15)	(13)	(11)
Poor physical health	799	1,228	2,315	510	308	249	342	630	29	21	114	35	114	44	15	17	6
Unexplained	107	-457	-1339	-1250	-986	734	897	575	52	442	(36)	314	30	22	(385)	(205)	(275)

#### Outpatient expenditures

Adults from the high-income households had higher annual per-person outpatient care expenditures than either adults from the poor-income or low-income households. However, in decomposition analysis the model predicted higher outpatient expenditures for adults from the poor-income and low-income households than the high-income households. Thus, variables included in the regression analyses could not explain the higher outpatient medical expenditure in adults from the high-income households.

#### Prescription drug expenditures

Decomposition analysis was able to explain 63% of the higher expenditures on prescription drugs for adults from the poor-income versus the low-income households, 64% for adults from the poor-income versus middle-income households, and 72% for adults from the poor-income versus high-income households. Medicaid–Medicare dual coverage, poor physical health status, and disability were significant factors for all 3 comparisons. In addition, the higher proportion of the population aged 55 to 64 years from poor-income households was associated with higher prescription expenditure when compared with adults aged 55 to 64 years from low-income and middle-income households. Offsetting factors included a lower proportion of White population among adults from poor-income households compared with adults from high-income households, a lower Medicare-only insurance population from poor-income households compared with adults from the low-income and middle-income households, and a higher uninsured population from poor-income households compared with adults from the middle-income and high-income households.

#### Home health care services expenditures

Decomposition analysis explained 95% of the difference between expenditures of adults from the poor-income and low-income households and 77% between adults from the poor-income and the middle-income households, and it overestimated the difference in expenditures between adults from the poor-income versus high-income households by 2%. The top elevating factors to explain the difference between adults from poor-income and both the low-income and middle-income households included a higher Medicaid–Medicare dual coverage rate, a higher disability rate, a lower Medicare-only rate, and a higher rate of perceived poor mental health. In contrast, a higher proportion of people aged 55 to 64 from the poor-income households was an offsetting factor. Higher Medicaid–Medicare dual coverage and disability rates were the 2 elevating factors to explain the difference in home health care services expenditure between adults from poor-income households and adults from high-income households.

For adults from low-income versus the middle-income and high-income households, 61% and 93%, respectively, of the differences were explained by the factors included in our model. The factors significant in explaining the differences were similar between adults from the poor-income and high-income households, except that a higher rate of poor physical health was also a significant elevating factor.

#### Emergency department expenditures

Our model explained 74% of the difference in emergency department expenditures between adults from the poor-income and high-income households. Higher disability rates and higher rates of reported poor mental and physical health were significant elevating factors, while higher Medicaid–Medicare dual coverage rate was the only significant offsetting factor.

#### Other medical expenditures

Our model explained only a small portion of the difference in other medical expenditures among adults from the poor-income, low-income, and middle-income households versus the high-income households, indicating that factors not included in the model may play a more important role than the factors included in the model regarding group differences.

### Sensitivity analyses

In US adults without diabetes, annual per-person medical expenditures ranged from $5,769 (middle income) to $6,663 (high), significantly lower than for those with diabetes in the same income groups. Unlike the descending order in total medical expenditure (poor, low, high, middle) in the population with diabetes, the order is high, poor, low, middle in the population without diabetes. Total medical expenditure was significantly higher in adults from the poor-income ($553) and high-income ($894) households compared with middle-income group, with no significant differences for other pairs. The annual per-person expenditure gap was $894 between the income groups with the highest and lowest expenditure among the population without diabetes, versus $4,141 for the population with diabetes. The difference between adults from the middle-income and high-income households was mainly from higher outpatient expenditures in adults from the high-income households, while the difference between adults from middle-income and poor-income households was mainly from higher inpatient expenditure in the poor-income group in the population without diabetes. But in the population with diabetes, the difference in annual per person costs was from prescription and home health care services expenditure differences between the poor-income group and the other 3 income groups.

Prescription and home health care services accounted for more than 20% of expenditure differences (prescription: poor vs low 67%, poor vs middle 60%, poor vs high 67%; home health: 35%, 45%, 65%, respectively). Usage and per-unit expenditure analyses compared prescription drugs and home health care services between adults from the poor-income households and others. For prescription drugs, the unit expenditure per refill was significantly higher for adults from the high-income households compared with adults from the other 3 income level households; unit expenditure per refill was higher in adults from the poor-income than the low-income households. The total number of prescription refills in a year decreased as income level increased, and the differences were significant between all comparison pairs.

The expenditure per home health care visit was highest in adults from the high-income households, followed by low-income, middle-income, and poor-income households. Expenditure per home health care visit was significantly lower for adults from poor-income households compared with adults from low-income and high-income households. The total number of home health care services in a year decreased as income level increased, and the differences were significant for all comparison pairs.

To better understand the relationship between income and health, we conducted an additional analysis comparing comorbidity profiles across income levels. Most chronic conditions — such as high blood pressure, arthritis, high cholesterol, asthma, coronary heart disease, myocardial infarction, and stroke — were more prevalent among adults from lower-income households, with significant differences observed between adults from the poor-income or low-income households and adults from the high-income households. Overall, these findings are consistent with our main results, which indicate poorer physical health among adults from the lowest-income households.

## Discussion

We examined differences in medical expenditures among US adults with diabetes by income level and found that adults from the lowest income (poor) households faced significantly higher expenditures — $2,928 to $4,141 or 15% to 22% more than adults from higher income households. This discrepancy primarily stems from spending on prescription drugs and home health care services. Higher disability rates, poor physical health status, and Medicaid–Medicare dual coverage were the most important factors explaining the difference between adults from the lowest income households and other income level households. Our study is the first to describe expenditure differences by income level and to explore the associated factors in US adults with diabetes. Our cost estimates by income group could serve as a measure for planning the budget needed for social programs targeting low-income people. By identifying cost sources and factors behind expenditure gaps, our findings may help inform efforts to ease the financial burden of diabetes across income levels.

Our results align with studies focused on the general US population, showing that people from families with lower incomes use more health care services and face higher medical costs (19,20), primarily due to poorer health status and higher rates of disability (19–21). Poor health and disabilities increase care needs but can also limit work, reducing income and worsening financial strain. In our study, the rate of disability in adults from the poor-income households was nearly twice as high as for adults from the high-income households, which explains a large proportion of higher medical expenditures among adults from the poorest income households (22). Previous studies have also found that disability status is strongly associated with elevated medical expenditures in people with diabetes (23). Despite the availability of various government benefit programs, many people still face long-term income loss due to disability (24). Diabetes increases the risk of physical disability by 50% to 90% (25). Preventing disability among people with diabetes could therefore offer a twofold benefit: improving health outcomes and preserving peoples’ ability to maintain stable employment and income, thus reducing health care needs.

Dual Medicaid–Medicare coverage status was significantly associated with the income-related medical expenditure difference in our study, highlighting the role of the dual-coverage policy in supporting people with greater needs. Dually covered beneficiaries have low incomes and are either elderly or have long-term disabilities, leading to higher medical care needs and a larger share of expenditure from both programs (26,27). Among the Medicaid–Medicare dually covered population with diabetes, higher rates of diabetes-related complications and comorbidities were observed compared with people with other insurance types. Dual coverage helped address barriers to health care access (28) and was shown to be effective in meeting the complex health needs of eligible people.

Diabetes influenced both the magnitude and pattern of income-related differences in medical expenditure. People without diabetes had 34% to 42% lower annual per-person medical expenditures than those with diabetes (Table 2). Furthermore, the income-related expenditure gap was much greater among people with diabetes. The difference in medical expenditures between income groups was $905 (13%) for those without diabetes compared with $4,141 (22%) for those with diabetes (Table 2).

Our findings suggest that efforts to improve health status and prevent disability in the lowest income group could yield financial benefits due to their high medical expenditures. For instance, studies evaluating the health and economic impact of diabetes prevention programs in Medicaid populations showed that such programs were cost-effective, could result in cost savings over a 25-year horizon, and could improve health equity (29). Additionally, as diabetes is a strong risk factor for disability, preventing diabetes at a population level would also be effective in reducing disability rates, further lowering medical expenditures in the lowest income group. Among people with diagnosed diabetes, management education programs aimed at reducing complications have been particularly cost-effective and even cost-saving, especially for low-income patients (30,31).

### Limitations

This study has several limitations. First, MEPS is limited to the civilian noninstitutionalized population, so expenditures for people in long-term care for disability-related reasons were not included, potentially underestimating medical expenditure differences. Second, results from our decomposition analyses should be interpreted with caution, as the associations between factors and expenditures are not causal. Medical expenditures may be affected by other factors not included in our model, such as health insurance policies, purchaser options, national and state policies, and drug patents. Third, self-reported health status may not accurately reflect actual health care needs. The large portion of the expenditure difference explained by Medicaid–Medicare dual coverage does not suggest that eliminating these benefits would reduce disparities; rather, dual coverage provides the financial means necessary to meet health care needs for the poor. Lastly, while we attempted to use specific comorbidities and complications as proxies, sample size limitations and missing data prevented further analysis.

### Conclusion

Significant differences in medical expenditures per person per year exist by income level among US adults with diabetes, with the poorest income group incurring 15% to 22% higher expenditures than higher income groups. Higher spending on prescription drugs and home health care services were the 2 main drivers. Higher disability rates, poor reported physical health status, and Medicaid–Medicare dual coverage largely explain these expenditure differences. Programs aimed at improving health status and preventing disability in low-income populations with diabetes may help reduce medical expenditure disparities between income groups in the US.
